# Enhancement of Early Cervical Cancer Diagnosis with Epithelial Layer Analysis of Fluorescence Lifetime Images

**DOI:** 10.1371/journal.pone.0125706

**Published:** 2015-05-12

**Authors:** Jun Gu, Chit Yaw Fu, Beng Koon Ng, Lin Bo Liu, Soo Kim Lim-Tan, Caroline Guat Lay Lee

**Affiliations:** 1 Optimus, Photonics Center of Excellence, School of Electrical and Electronic Engineering, Nanyang Technological University, Singapore, Singapore; 2 KK Women’s and Children’s Hospital, Singapore, Singapore; 3 Department of Biochemistry, Yong Loo Lin School of Medicine, National University of Singapore, Singapore, Singapore; 4 National Cancer Center, Singapore, Singapore; 5 Duke-NUS Graduate Medical School, Singapore, Singapore; Brigham and Women's Hospital/Harvard Medical School, UNITED STATES

## Abstract

This work reports the use of layer analysis to aid the fluorescence lifetime diagnosis of cervical intraepithelial neoplasia (CIN) from H&E stained cervical tissue sections. The mean and standard deviation of lifetimes in single region of interest (ROI) of cervical epithelium were previously shown to correlate to the gold standard histopathological classification of early cervical cancer. These previously defined single ROIs were evenly divided into layers for analysis. A 10-layer model revealed a steady increase in fluorescence lifetime from the inner to the outer epithelial layers of healthy tissue sections, suggesting a close association with cellular maturity. The shorter lifetime and minimal lifetime increase towards the epithelial surface of CIN-affected regions are in good agreement with the absence of cellular maturation in CIN. Mean layer lifetimes in the top-half cervical epithelium were used as feature vectors for extreme learning machine (ELM) classifier discriminations. It was found that the proposed layer analysis technique greatly improves the sensitivity and specificity to 94.6% and 84.3%, respectively, which can better supplement the traditional gold standard cervical histopathological examinations.

## Introduction

Every year approximately 300,000 women die from cervical cancer with approximately 510,000 new cases diagnosed [1, 2]. Preceding the invasive cancer is the slow-growing cervical intraepithelial neoplasia (CIN) phase in which early detection and treatment is feasible. Consequently, many cervical screening protocols have been implemented to detect the pre-malignant cervical changes and the cervical cancer associated deaths have significantly declined.

The primary screening method for early cervical cancer is the Papanicolaou (Pap) smear test. Cells from cervix wall with suspected signs of neoplasia are collected for microscopic examination. However, the Pap test is complicated and labor-intensive and it shows a concurrent low sensitivity (≤58%) and specificity (69%) [3, 4], implying the need for repeated tests to reduce the false-negatives. In addition, colposcopy has to be subsequently conducted to confirm the abnormality. In expert hands colposcopy could achieve excellent sensitivity (>90%) but low specificity (<50%), which further requires a directed biopsy for definitive diagnosis [5]. The thinly-sliced biopsy is stained with haematoxylin and eosin (H&E) to enhance the visual contrast for histopathological evaluations. Haematoxylin stains the nuclei purple while eosin stains the intracellular and extracellular protein pink. Morphological features, such as cell shape, nuclear size and nuclear-to-cytoplasm (N/C) ratio are commonly used as diagnostic criteria by histopathologists to identify abnormal cells [6, 7].

Cervical precancerous conditions are described by a well-defined three-tier grading system named cervical intraepithelial neoplasia (CIN) [8, 9]. CINs are classified in grades, namely CIN1 (mild dysplasia), CIN2 (moderate dysplasia) and CIN3 (severe dysplasia) [10]. The three grades of CIN refer to the thickness of the epithelium affected by abnormal cells [11]. When the basal third of the epithelium is replaced by abnormal cells, the tissue is histologically defined as CIN1. CIN2 is used when the middle and basal third epithelium is occupied by neoplastic cells while the complete replacement of abnormal neoplastic cells defines CIN3. In most hospitals, identifying the CIN grade of H&E stained tissue sections is regarded as the gold standard for cervical cancer diagnosis [12, 13]. However, great variability can exist in diagnostic results because of the labor-intensive procedure and the subjective interpretation involved [14]. In addition, implementation of this approach requires extensive infrastructure, personnel and economic resources. Women, especially those from regions with low-resource settings, may not have access to these screening programs [15]. Therefore, new automated imaging modalities that allow for non-invasive and more accurate evaluation of cervical carcinoma would overcome these limitations and greatly improve the prevention of cervical cancer.

Several optical techniques have been developed to aid the in vivo and in vitro diagnosis of cervical precancer [5, 16–21]. These techniques differ from the gold standard diagnosis in how tissues are processed. In particular, reflectance spectroscopy, elastic scattering spectroscopy and fluorescence spectroscopy capable of characterizing morphological and biochemical information have been used for diagnostic purposes. The use of H&E dye as a contrast agent in non-linear imaging and fluorescence imaging has been reported elsewhere [22–24], but its latent diagnostic value for cancer detection has yet to be fully explored. Our previous study [25] on standard H&E stained cervical tissues showed that fluorescence lifetime imaging (FLIM) was particularly useful for detecting abnormal biochemical changes related to CIN. The FLIM technique has the advantage of being sensitive to biochemical changes in tissue micro-environment which correlates to tissue pathology [26, 27]. Furthermore, H&E stained cervical tissue sections generate much stronger fluorescence emission as compared to the low autofluorescence from unstained tissues [25]. The diagnostic capability is thus enhanced by the more precise calculation of fluorescence lifetime values derived from larger number of emission photons [28]. In addition, illumination variations and staining artifacts are eliminated since fluorescence lifetime is independent of excitation power and fluorophore concentration [29].

The gold standard diagnosis of cervical precancer (CIN) is based on the proportion of epithelium affected by neoplasia, suggesting that diagnostic information exists in the layered structures of cervical tissue sections. In this work, the diagnostic value in the layered structures of H&E stained cervical tissue sections was investigated with FLIM. H&E stained cervical tissue sections classified into categories of normal and cervical intraepithelial neoplasia (CIN1, CIN2, CIN3) were used for fluorescence lifetime imaging and analysis. Lifetime values were first analyzed on a 3-layer model following the standard (CIN) diagnosis. Subsequently, epithelial regions of these cervical tissue sections were divided into 10 equal layers and analyzed. The 10-layer model is based on the findings that epithelium is generally composed of approximately 10 layers [11]. The basal layer which adheres to the basement membrane is one cell thick. Above this layer are the parabasal cells which are two to three cells in thickness. These parabasal cells mature and form the upper intermediate and superficial layer. Such layer division was applied to assess the diagnostic values of fluorescence lifetime data measured from each cellular layer. Feature vectors comprising lifetimes from different layers of the epithelial tissues were fed into a neural network extreme learning machine (ELM) classifier [30, 31] for discriminations between normal and CIN. ELM classifier was selected due to its ability to detect complex trends with good generalization performance at extremely fast learning speed.

This work showed that there exist an optimal number of cervical epithelial layers to consider in the analysis at which the diagnostic accuracy is maximized. The accuracy here was evaluated in terms of sensitivity and specificity. Interestingly, we identified the top half epithelium as the effective zone for FLIM diagnosis with concurrently high sensitivity (94.6%) and specificity (84.3%). The obtained average of sensitivity and specificity is about 1.5 times better than those in whole-epithelium and 3-layer epithelium models. The proposed layer analysis of FLIM diagnosis can further aid and complement traditional histopathological examinations of cervical diseases with better diagnostic accuracy.

## Materials and Methods

### Samples

A total of 32 H&E stained cervical tissue sections of 32 patients from the KK Women's and Children's Hospital (KKH), Singapore were used for the analysis carried out in this study. Each chosen section has clear stratified layers that facilitate layer analysis to be made for this pilot study. These tissue sections were pathologically examined by a senior consultant from the KKH and the identified regions in the epithelium were classified as normal, CIN1, CIN2 and CIN3. The sample set includes 10 normal, 8 CIN1, 6 CIN2 and 8 CIN3 cervical tissue sections. This study was reviewed and approved by the local ethics committee (KK Women's and Children's Hospital (KKH), Singapore) and Institutional Review Board (CIRB 2010/745/C). Written consent was obtained from participants for the use of information in medical studies.

### Imaging Protocol and Lifetime Calculation

The imaging protocol was identical to that used in our previous work [25]. White light microscopy was initially used to locate the identified regions-of-interest and a time-resolved fluorescence measurement system, comprising a confocal laser scanning microscope (LSM 510, Carl Zeiss, Germany) and a time-correlated single photon counting (TCSPC) system, was used for fluorescence lifetime imaging. The illumination light source is a femtosecond Ti: Sapphire laser (Coherent Mira 900, 76 MHz, 200 fs) operated at a wavelength of 760 nm. A 20× objective lens (Fluar, Carl Zeiss, NA = 0.75) was used to focus the excitation laser beam to the tissue sample and to collect the fluorescence emission. Fluorescence lifetime images were captured in a scanning time window of 10 ns with a temporal resolution of approximately 39 ps. The acquisition time for an image comprising of 256×256 pixels was 60 s. The image has a pixel size of approximately 1.8 μm.

The fluorescence lifetime *τ* is defined as the time that the emission fluorescence intensity decreases to 1/e of its initial value after a fluorophore is excited by a light pulse. The decay mechanism of the fluorescence emission intensity *I*(t) is described by [32]:

$$\frac{dI(t)}{dt}=-\frac{1}{\tau }I(t)$$ (1)

where τ = 1/(Kr+Knr), Kr and Knr are the radiative and non-radiative transition rates, respectively, and *t* is the time elapse since light excitation. The fluorescence lifetime was calculated with the commercial software SPCImage (Becker & Hickl, Germany). The threshold value for curve fitting was set to 50 photons so that dark pixels with cumulative photon counts of less than 50 were not included in lifetime calculation. The instrument response function (IRF) was estimated based on the rising edge of the decay curve and a double exponential model was used for a good curve fitting with its reduced chi-square value (χ2) approaching unity. The double exponential model of the fluorescence decay characteristic is expressed as:

$$I(t)=a_{1}\exp(-\frac{t}{\tau_{1}})+a_{2}\exp(-\frac{t}{\tau_{2}})$$ (2)

where τ_1_ and τ_2_ are the fluorescence lifetimes, and a_1_ and a_2_ are the fractional contribution of each lifetime component. Lifetime values, τ_1_ and τ_2_, were obtained using nonlinear least-squares (NLLS) algorithm [33]. Our previous work has shown that the longer lifetime component τ_2_ distribution yield the notable diagnostic contrast among various cervical cancer stages [25]. On the other hand, the shorter lifetime component τ_1_ with a typical value of ~30 ps and was attributed to instrumental response time and has no correlation to tissue pathology. A false-colored lifetime image consisting of 256×256 pixels was generated by mapping a specific color to the lifetime value of τ_2_ at each pixel.

### Cervical Epithelial Layer Definition and Feature Vectors

**Fig 1:**
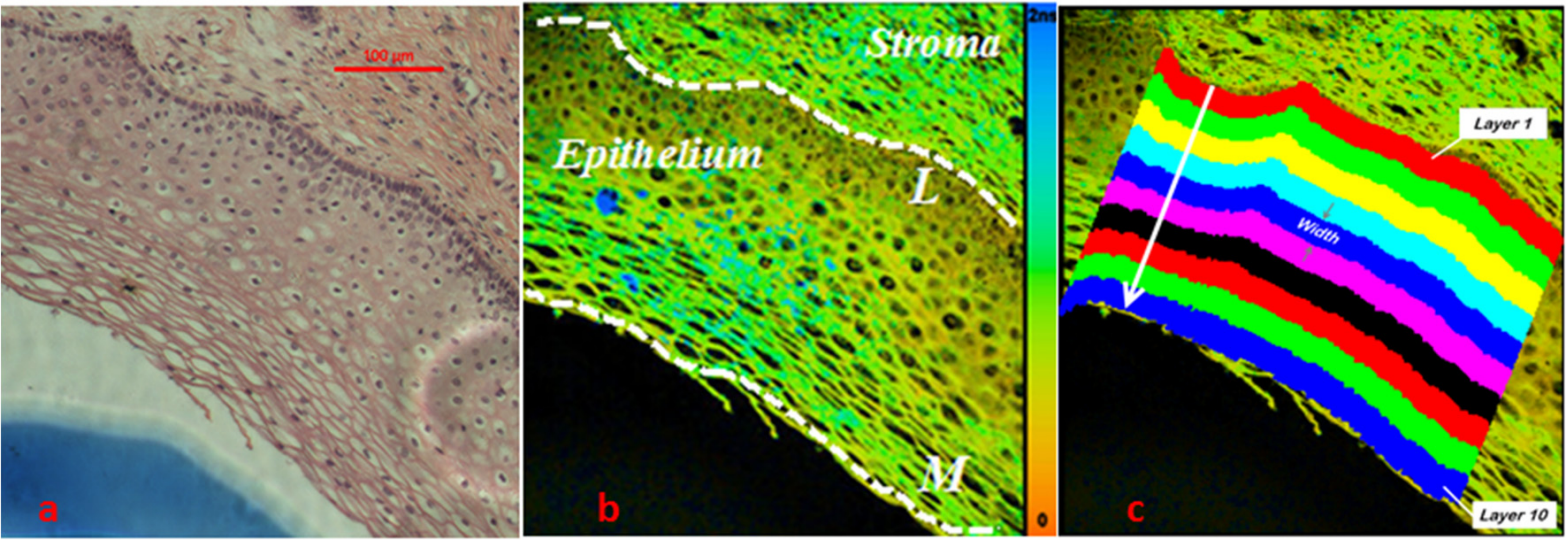
Division of a typical H&E stained cervical tissue section epithelium into 10 layers and the corresponding ROI defined in the fluorescence lifetime image. (a) White light image of the H&E stained tissue section (b) False color fluorescence lifetime image with scale bar from 0 to 2 ns. Basement membrane was marked out by white dashed line *L* and epithelium surface was delineated by white dashed line *M*. (c) Pixels in each divided layer were obtained by moving basement membrane pixels in the direction perpendicular to the basement membrane towards the epithelial surface. Layers are numbered incrementally from 1 to 10. Pixels in each divided layer constitute the ROI.

Epithelium regions in cervical lifetime images were divided into layers to analyze the characteristics of the lifetime component τ_2_ along the epithelial growth direction. Cells in the epithelium grow and differentiate progressively in the direction towards the epithelial surface. The basement membrane, a thin and noncellular region between the epithelium and the neighboring stroma, forms a barrier to downward epithelial growth and is only breached if epithelium undergoes malignant transformation [34]. The structural difference in epithelium and stroma makes them clearly distinguished in white-light image and fluorescence lifetime image as shown in Fig 1. The basement membrane is marked by a white dashed line L while the epithelial surface is outlined with white dashed line M (Fig 1B). Epithelial thickness is defined as the distance from a point on the epithelial surface to the basement membrane along the direction normal to the basement membrane. In our measurement the epithelium generally has a thickness of 260 ± 60 μm on gross examination which is consistent with literature data of 200–500 μm [35]. The epithelium was divided into 3 and 10 equal thickness layers, respectively along the direction of tissue growth [34] for lifetime analysis. The ROI in the epithelium for lifetime analysis is defined with equal width of each divided layer. Fig 1C shows a typical cervical tissue section epithelium divided into 10 layers. Layer 1 refers to the basal layer while layer 10 indicates the superficial layer. The 3-layer model analysis is of great interest for diagnostic comparison due to the classic definition of CIN, which is based on the thickness of epithelium covered by neoplastic cells. On the other hand, the 10-layer model is used to approximate the biological structure of a cervical epithelium, with its cellular maturity increasing towards the epithelial surface. Thus, the 10-layer analysis model has the advantage of evaluating the CIN cells as a function of their maturity level. Finally, the mean (*μ*) and standard deviation (*σ*) of lifetime in each divided layer was calculated for tissue classification.

### Extreme Learning Machine Classification Algorithm

Discrimination between normal and precancerous (CIN1, CIN2, CIN3) cervical tissues were performed using a neural network ELM classifier. As compared to other conventional neural network classifiers such as the support vector machines (SVMs) and back-propagation (BP) method, ELM has a better generalization performance with greater learning speed because all weights in the hidden nodes are randomly generated without the need to be iteratively tuned [36]. The ELM learning algorithm is implemented as follows. Consider a training set Ψ = {(***x***_i_, ***t***_i_) | ***x***_i_ = [*x*_i1_, *x*_i2_, …, *x*_in_]^T^ ∊ *R* ^n^, ***t***_i_ = [*t*_i1_, *t*_i2_, …, *t*_im_] ∊ *R* ^m^, i = 1, …, *N*} where ***x***_i_ and ***t***_i_ specify the input lifetime feature vectors and the associated output target describing the pathological state, and *N* represents the number of samples in the training set. The hidden layer output matrix **H** and the output weight **β** of the neural network are calculated in the following steps:

1. Randomly assign input weight vectors **a**_i_ and hidden node bias b_i_, i = 1,…, *L* where *L* is the number of hidden nodes. *L* value was adjusted so that maximum discrimination accuracy was achieved in this study.
2. Calculate the output function of each hidden node g(**a**_i_, b_i_, **x**), i = 1, …, N and the hidden layer output matrix **H** = [*g*(**a**_1_, b_1_, ***x***), …, *g*(**a**_L_, b_*L*_, ***x***)]. Here, *g*(**a**_i_, b_i_, ***x***) = 1/(1+exp[-(**a**_**i ∙**_ ***x*** +b_i_)]).
3. Determine the output weight, **β** = **H** ^+^ **T** where **H** ^+^ is the Moore-Penrose generalized inverse of the hidden layer output matrix **H** and **H** satisfies the equation **Hβ** = ***t***, where **β** = [β_1_, …, β_*L*_]^T^.

In this work, *n* specifies the total number of feature components in each sample. In the 3-layer analysis, *μ* and *σ* from layer 1 to layer 3 constitute a total of 6 feature components and thus *n* = 6. Meanwhile, *m* indicates the two possible pathological states of a cervical tissue sample, e.g. normal or precancerous, and thus *m* = 2.

In ELM, the output weight **β** of the neural network is first calculated based on the input feature vectors and their known target output from the training data set. This output weight **β** was then used to determine the output of the feature vectors in the testing data set and compared to their known target output to derive the diagnostic accuracy.

Cross-validation technique was applied to achieve unbiased selection of training data and testing data. Basically, it randomly splits the whole dataset into training dataset and testing dataset. Training data and testing data sets of equal sizes are used to maximize the accuracy of ELM algorithm in this case. Hence, feature vectors from 16 randomly selected samples were chosen as training data while feature vectors from the remaining 16 samples were used for testing. A total of 1000 random sets of training and testing data were generated for computation to reduce classification bias. For each set of randomly selected training data and testing data, ELM is applied to distinguish between histologically identified normal and CIN samples. The diagnostic accuracy (sensitivity and specificity) for each set of training data and testing data is then calculated by comparing the ELM result with the histological identification. Finally, the mean sensitivity and specificity from the 1000 sets of data were computed accordingly for comparison.

## Results

### Lifetime Distribution in Whole Cervical Epithelium

Fig 1A shows the white-light microscopic image of a typical H&E stained normal cervical tissue and Fig 1B shows the corresponding fluorescence lifetime image. A comparison between the two images reveals that additional biochemical contrast could potentially be extracted from the lifetime image to characterize cervical tissues. It can be seen that the epithelium region is made up of multiple layers of closely-packed cells. The stroma region, which comprises of fibroblasts, smooth muscle cells and collagen [37], is quite distinct. The boundary between the two regions can easily be identified as depicted in Fig 1B.

The cell-rich epithelium region was specifically investigated and diagnosed with FLIM technique since cervical cancers mostly originate from the epithelium [38]. The diagnostic value of mean (*μ*) and standard deviation (*σ*) of τ_2_ distribution in the whole epithelium is first explored for comparison with layer analysis. Normal samples were found to have larger lifetimes in the range from 670 ps to 1000 ps while precancerous tissues have smaller lifetimes distributed from 450 ps to 850 ps. On the other hand, normal samples have lifetime *σ* values distributed between 150 ps and 200 ps while precancerous samples have lifetime *σ* values in the range of 170 ps to 280 ps. This allows a discrimination to be made between normal and precancerous cervical samples based on our previous work [25]. The diagnostic accuracy of whole-epithelium analysis was assessed with ELM classifier, giving an averaged sensitivity of 62.2% and specificity of 52.3%.

### Three-Layer Analysis of Cervical Epithelium Lifetime

**Fig 2:**
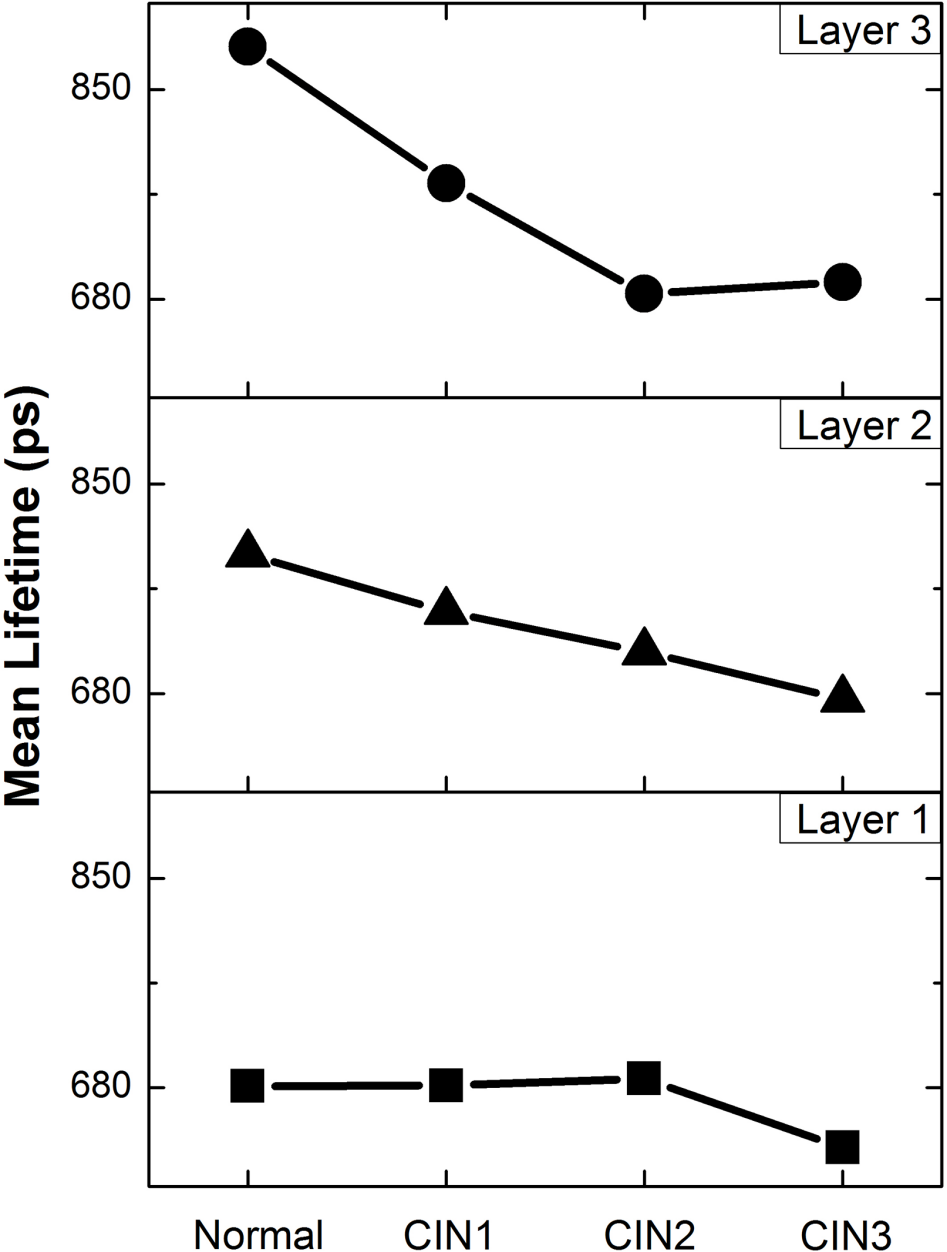
Distribution profiles of mean τ_2_ in divided layer 1 (■), layer 2 (▲) and layer 3 (●) of epithelium against pathological states of Normal, CIN1, CIN2 and CIN3. Here, the mean τ_**2**_ for each pathological state was calculated from the sample pool which includes 10 normal, 8 CIN1, 6 CIN2 and 8 CIN3 cervical tissue sections and the averaged relative standard deviation (RSD) was calculated to be 15% for all categories. More details regarding error bars are elucidated in S3 Fig.

Each cervical epithelium was divided into 3 layers and the associated τ_2_ distribution profiles were extracted for each layer and compared against the pathological stages (as shown in Fig 2). A consistent trend of τ_2_ shortening can be observed as cervical tissue progresses from normal to CIN3, except for layer 1, where τ_2_ only shortens when the disease stage progresses from CIN2 to CIN3. The lifetime difference between normal and CIN 3 samples was estimated to be 50 ps at layer 1, 80 ps at layer 2 and 100 ps at layer 3. Mean lifetime of τ_2_ in each divided layers was used to form a 3-dimensional feature vectors for ELM classification. The ELM classification method gives an improved sensitivity of 81.1% and a decreased specificity of 39.6%. Standard deviation of τ_2_ distribution was also investigated, but its varying trends (S1 Fig) did not contribute to any significant improvement in diagnosis and are therefore considered uncorrelated to tissue pathology. The results indicate that feature vectors comprising only the mean τ_2_ is sufficient to achieve the same diagnostic sensitivity and specificity.

## Ten-Layer Analysis of Cervical Epithelium Lifetime

**Fig 3:**
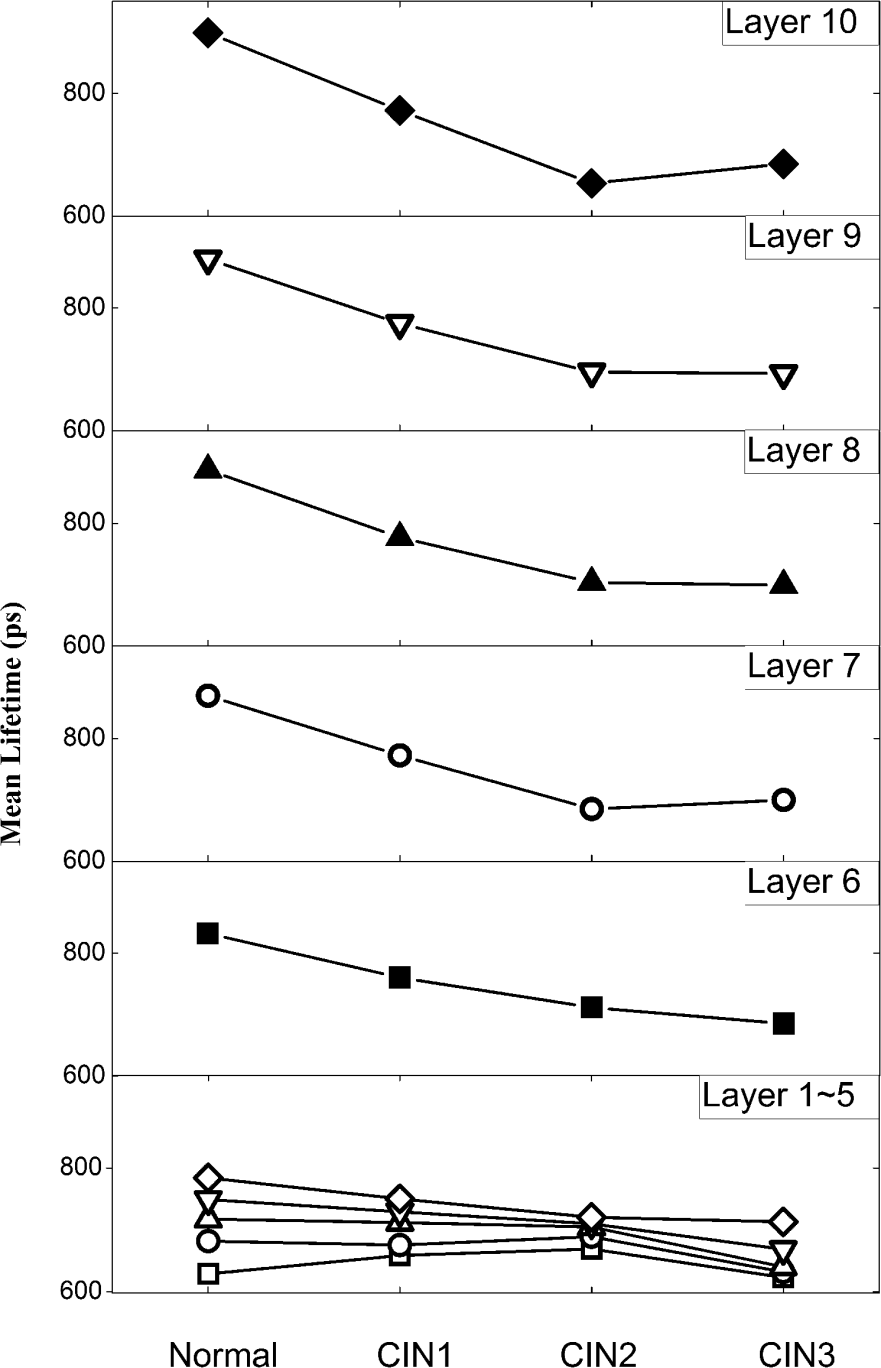
Distribution of mean lifetime τ_2_ in lower half layers (□-layer 1, ◯-layer 2, △-layer 3, ▽-layer 4, ◇-layer 5) and top half layers (6–10) of epithelium as tissues progress from normal to various CIN grades. Here, the mean τ_**2**_ for each pathological state was calculated from the sample pool which includes 10 normal, 8 CIN1, 6 CIN2 and 8 CIN3 cervical tissue sections and the averaged relative standard deviation (RSD) was calculated to be 20% for all categories. More details regarding error bars are clarified in S4 Fig.

The distribution of τ_2_ in the 10 divided layers of each sample were analyzed for the various histological stages and are presented in Fig 3. Distributions of τ_2_ in the first 5 layers (1–5) among normal and CIN samples were first compared. Despite a consistent shortening of τ_2_ in layers (3–5) as CIN progresses, the value of mean τ_2_ change is below 70 ps when comparing normal epithelium with CIN 3 samples. By contrast, the mean τ_2_ distribution from layer 6 to 10 shows greater variation across different pathological stages, with normal tissues exhibiting the longest lifetime. The mean τ_2_ values in layer 6 to 10 were observed to consistently decrease as the tissues progress to the precancerous stages. When comparison is made from layer 6 to layer 10, the smallest lifetime variation of ~147ps is found at layer 6 while the largest change of ~245ps occurs in layer 10. Similar to the 3-layer epithelium analysis, standard deviation *σ* of lifetime τ_2_, with poor pathological correlation (S2 Fig), was not included into the feature vectors for tissue classification.

**Fig 4:**
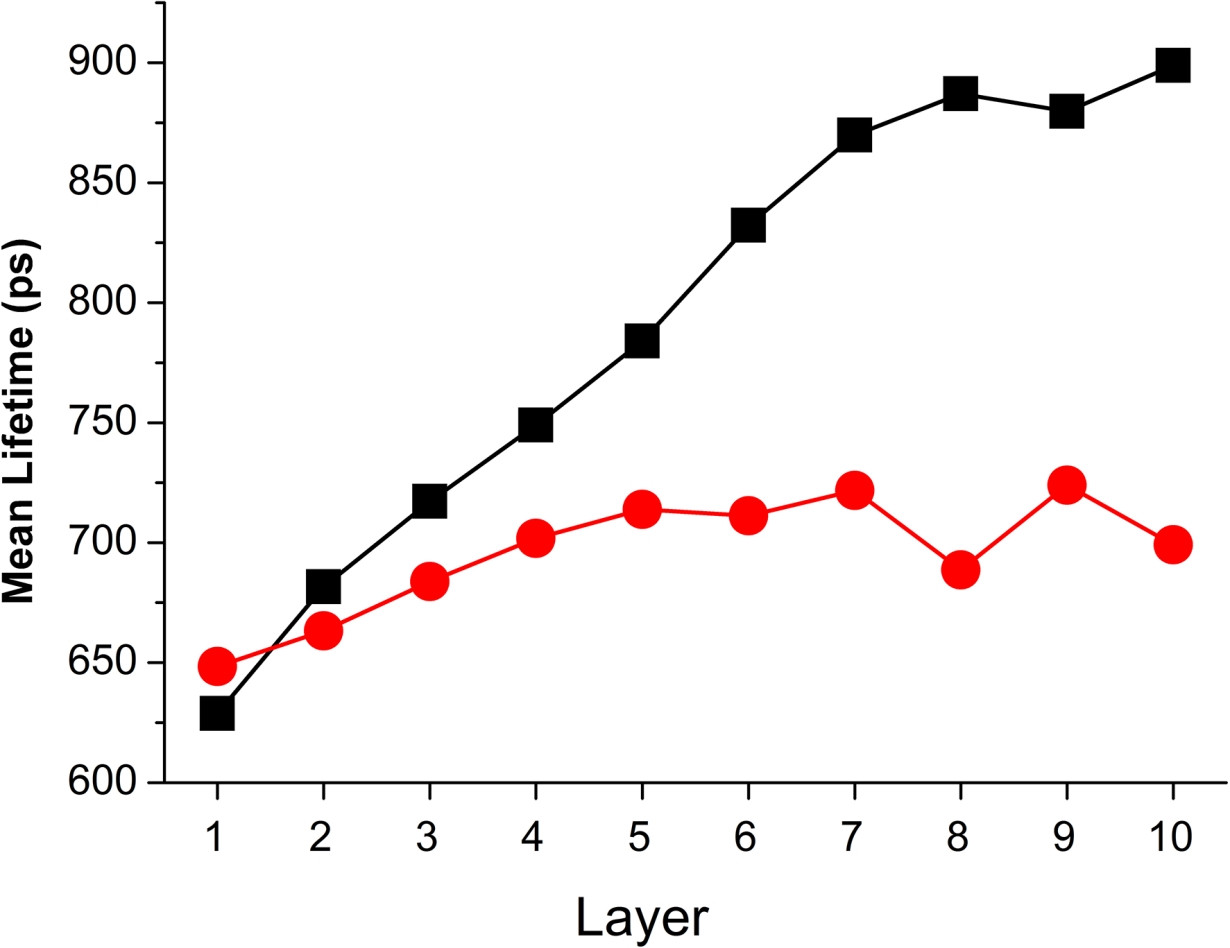
Distribution of mean τ_2_ in all divided 10 layers for normal (black square) and precancerous (red circle) samples. Here, the mean τ_**2**_ for each pathological state was calculated from the sample pool which includes 10 normal, 8 CIN1, 6 CIN2 and 8 CIN3 cervical tissue sections and the averaged relative standard deviation (RSD) was calculated to be 18% for all categories. More details regarding error bars are clarified in S5 Fig.

The mean τ_2_ values of normal and precancerous (CIN1, CIN2, CIN3) samples in each layer were also plotted in Fig 4 for comparison. Within each layer, varying differences between normal and precancerous samples was observed, thereby providing differing degree of diagnostic information. It is desirable to have a suitable combination of mean τ_2_ values to improve the classification performance [39].

**Fig 5:**
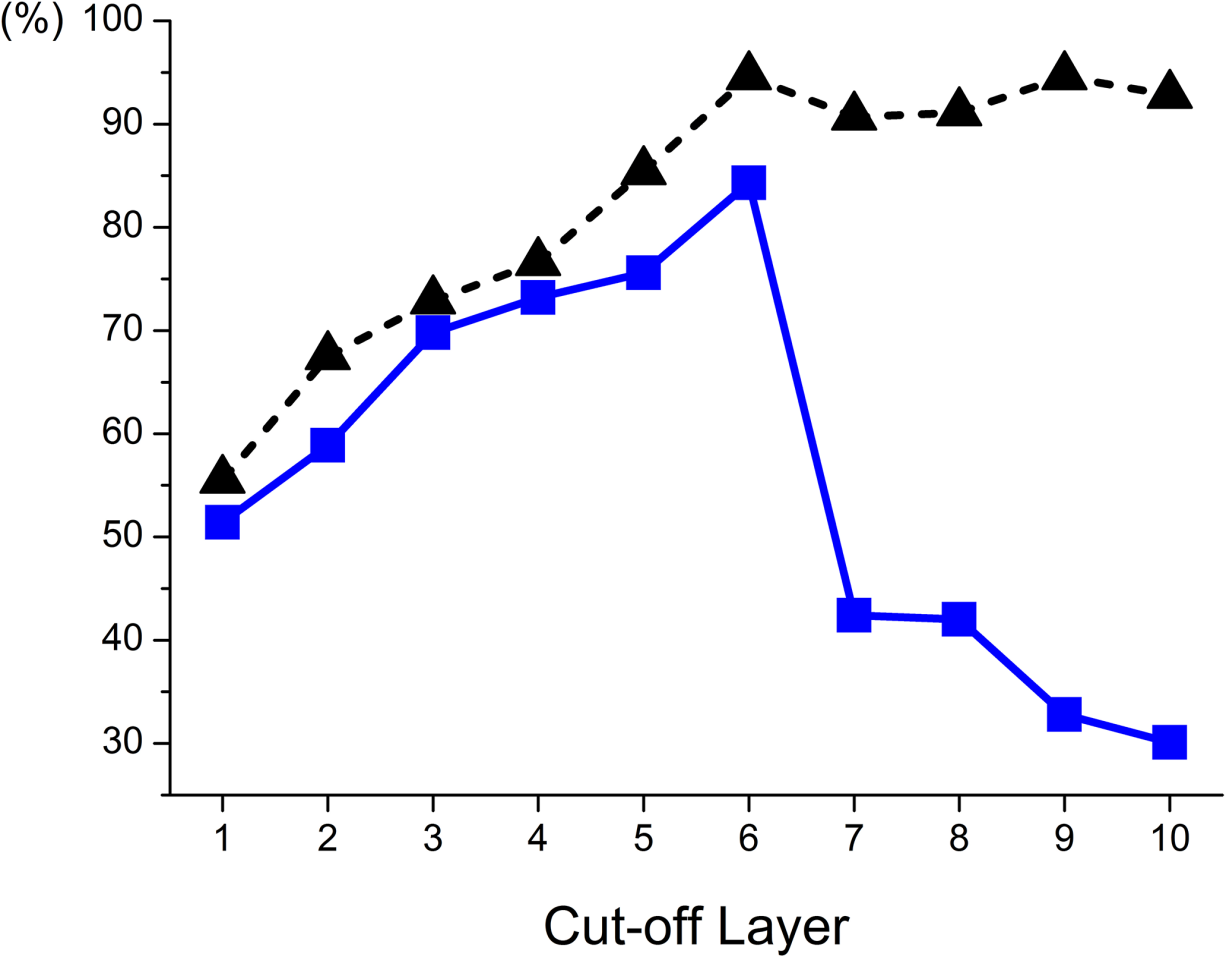
Variation in ELM diagnostic sensitivity (black triangle) and specificity (blue square) as feature vectors include mean τ_2_ values successively from epithelial surface (layer 10) to a lower layer as the cut-off layer. Optimum sensitivity (94.6%) and specificity (84.3%) were concurrently found at the cut-off layer of 6.

To investigate the optimal number of layers to take into account for maximum accuracy, feature vectors comprising mean τ_2_ values from successive layers, starting from layer 10, i.e. epithelial surface, to a lower layer (i.e layer 9) as the cut-off layer are studied. For instance, a typical set of feature vectors with cut-off layer at 9 is 2-dimensional containing mean τ_2_ values from layer 10 and layer 9 while cut-off layer at 6 results in a set of 5-dimensional feature vectors composed of mean τ_2_ from layer 10 to layer 6. ELM classifier was used to make classifications between normal and precancerous samples. The corresponding sensitivity and specificity as a function of the cut-off layer were calculated and shown in Fig 5. It can be seen from the plot that when the cut-off layer decreases from 10 to 6, the sensitivity increases slightly from 92.8% to 94.6% while the specificity rises sharply from 30.1% to 84.3%. Both the maximum sensitivity and specificity occur at the cut-off layer of 6. When the cut-off layer decreases from 5 to 1, both sensitivity and specificity decrease greatly and finally drops to a low value of 55.5% and 51.4%, respectively. The overall maximum sensitivity (94.6%) and specificity (84.3%) occurs when feature components τ_2_ from layers (6–10) were used for ELM classification.

The proposed epithelium layer analysis with neural network ELM classifier could achieve desirable discriminations between normal and precancerous cervical tissues when multiple feature vectors from mean τ_2_ values of layers (6–10) were used. The optimum sensitivity and specificity obtained are 94.6% and 84.3%, respectively. By contrast, whole epithelium analysis gave a sensitivity and specificity of 62.2% and 52.3% respectively. Three-layer analysis consistent with the generic definition of CIN achieves a sensitivity of 81.1% and specificity of 39.6%. Table 1 summarizes the diagnostic accuracies from the various analysis models used in this work. The 10-layer analysis suggests that the top half epithelium (layers 6–10) is the effective zone for FLIM diagnosis with concurrently high sensitivity and specificity. The average of obtained sensitivity and specificity (89.5%) is about 1.5 times those derived from whole-epithelium (57.3%) and three-layer (60.4%) analysis.

**Table 1:** ELM diagnostic sensitivity and specificity derived from various analysis models.

Analysis Model	Sensitivity	Specificity
**Whole epithelium**	62.2%	52.3%
**Three-Layer**	81.1%	39.6%
**Ten-Layer**	94.6%	84.3%

In addition, the computation time for data fitting and ELM classification was also calculated. The time used was halved as compared to the whole-epithelium analysis that involves data fitting for the whole epithelium. Lifetime data calculation by NLLS which is in the order of several minutes is computationally intensive and contributes most significantly in the overall processing time [40] while the ELM classification takes only tens of seconds with our system. Since only the top half epithelium is needed to derive mean τ_2_ values, the total processing time to calculate the lifetime data was decreased by two fold.

## Discussion

This is a pilot study investigating the use of layer analysis in FLIM images of H&E stained cervical tissue section epithelium for early cancer detection. A 3-layer epithelium model was first studied for comparison with the standard 3-tier histological grading system (CIN1, CIN2 and CIN3) which is based on the proportion of epithelial thickness affected by neoplasia. Lifetime diagnosis with a 10-layer epithelium model was also conducted and the resulting diagnostic accuracy was greatly improved. This model was investigated because it best represents the biological structure of cervical epithelium comprising 10 cellular layers with increasing maturity towards its surface. The classification result shown in Fig 5 identified the top-half epithelium (layers 6–10) as the most effective region for cervical epithelium diagnosis. It has been demonstrated in our previous work [25] that eosin is the contributing fluorophore which provides the molecular contrast. The improved diagnostic accuracy with the top-half epithelium can possibly be attributed to the prominent interplay between cellular maturity and eosin molecules. Normal cells are known to differ markedly from CIN cells in their ability to differentiate [41]. Normal cervical cells can differentiate to mature specialized cells as they grow upwards from the basement membrane to epithelial surface [42]. CIN cells, on the other hand, remain immature (undifferentiated) and proliferate vertically with increased abnormalities. It is evident in Fig 4 that mean τ_2_ of normal cells rises steadily from layer 1 to layer 10 where the cells are fully matured. By contrast, CIN cells show gradual increase in mean τ_2_ within the lower half epithelium and minimal change in the top-half layers. The significant difference in mean τ_2_ between normal and CIN cells, driven by their differing maturity, therefore constitutes the prominent diagnostic contrast in the top-half epithelium.

The shortening of τ_2_ is also observed in “normal” unaffected layers of CIN1 and CIN2, as defined by conventional grading system (see Fig 3). For instance, while CIN1 cells are in principle confined to the lower one-third epithelial layer according to standard diagnosis, shortening of τ_2_ (indicating precancer development) is still observed in the top-half epithelium. Hence, the shortening of τ_2_ is not solely attributed to the presence of immature CIN cells, but also other possible abnormalities starting to develop in the upper epithelial layers. First, while CIN cells are most prominent in the lower affected layers, abnormalities including increased mitotic activity and nuclear atypia can be present at all layers [43, 44]. Secondly, human papillomavirus (HPV) infection, a main cause of CIN [45], can result in koilocytosis, a condition where squamous epithelial cells have undergone structural changes in the top-half epithelium [46, 47].

Koilocytosis affects the cytoplasm of cells resulting in cellular changes and can be seen by identifying nuclei surrounded by tiny halos under white light microscopy. Cellular changes including morphological and biochemical changes are involved in the process of cervical dysplasia or even malignancy [11]. Therefore it is likely that early abnormalities occurring in top-half epithelium involves cellular changes that can contribute to lifetime change of eosin in the cytoplasm [25].

The incorporation of *σ* of τ_2_ in the feature vectors did not improve and may even deteriorate the diagnostic performance, suggesting that *σ* is not a good indicator of cellular abnormalities. This can be explained from the fact that *σ* depicts the spread the τ_2_ and thus the cellular homogeneity in each layer. In the 10-layer epithelium model, each layer would encompass cells of similar types and maturity [42], which would likely be independent of pathological states.

Dimensionality reduction of the raw input variables is an essential preprocessing step in the classification process [48]. It is necessary to keep the input features concise to reduce computational cost [49] and avoid performance degradation due to redundant and irrelevant features [50]. In this work, *σ* and τ_2_ of lower half epithelium were identified to be redundant and omitted from analysis.

The origins of the biochemical changes, and the associated eosin lifetime changes, as cervical cells become neoplastic warrants further investigation. Understanding the underlying mechanism to the biochemical changes in the cytoplasm would lead to more effective diagnosis and treatment of cervical cancer. The identification of the more useful diagnostic information in the top-half epithelium suggests that efforts in understanding the underlying biochemical process of cervical cancer development should concentrate on the top-half epithelium.

## Conclusion

A quantitative method based on the fluorescence lifetime imaging (FLIM) technique to aid the traditional gold standard histopathological diagnosis of cervical neoplasia was investigated. Fluorescence lifetime images of cervical epithelium were divided evenly into multiple layers in the tissue growth direction to study the diagnostic value of each layer. In 10-layer analysis, feature vectors comprising divided layer mean lifetime τ_2_ in the top-half epithelium (layer 6 to 10) were used for discrimination by a neural network ELM classifier. Averaged sensitivity and specificity of 94.6% and 84.3% were obtained when differentiating normal from precancerous (CIN1, CIN2, CIN3) tissues. On the other hand, in 3-layer analysis where feature vectors are from mean lifetime of τ_2_ in each of the three divided layers results in averaged sensitivity of 81.1% and specificity of 39.6%.

The proposed technique has the advantage of achieving a concurrently higher sensitivity and specificity as compared to the whole epithelium and 3-layer analysis. In addition, analyzing only the top-half of the cervical epithelium is computationally fast with significantly reduced lifetime calculation as redundant feature components were eliminated. The proposed method can provide more accurate and faster cervical diagnosis which can supplement traditional gold standard histopathological examinations. Furthermore, the reduction of feature vector size makes the ELM classifier a potentially more efficient tool when there is a need to analyze a large quantity of data in future applications.

## Supporting Information

S1 Fig Distribution of τ_2_ standard deviation in divided three layers among different pathological states of normal, CIN1, CIN2 and CIN3 tissues. (TIF)

S2 Fig Distribution of τ_2_ standard deviation in divided layers (from 1 to 10) among different pathological states of normal, CIN1, CIN2 and CIN3 tissues. (TIF)

S3 Fig Distribution profiles of mean τ_2_ in divided layer 1 (■), layer 2 (▲) and layer 3 (●) of epithelium against pathological states of Normal, CIN1, CIN2 and CIN3. (TIF)

S4 Fig Distribution of mean lifetime τ_2_ in lower half layers (□-layer 1, ◯-layer 2, △-layer 3, ▽-layer 4, ◇-layer 5) and top half layers (6–10) of epithelium as tissues progress from normal to various CIN grades. (TIF)

S5 FigDistribution of mean τ_2_ in all divided 10 layers for normal (■) and precancerous (●) samples.(TIF)

S6 Fig Original white-light image of a typical H&E stained cervical tissue section. (TIF)
