# Supplementary figures and images for: Enhancement of Early Cervical Cancer Diagnosis with Epithelial Layer Analysis of Fluorescence Lifetime Images
Source: PLoS One. 2015 May 12;10(5):e0125706. doi: 10.1371/journal.pone.0125706 (PMC4428628; doi:10.1371/journal.pone.0125706)

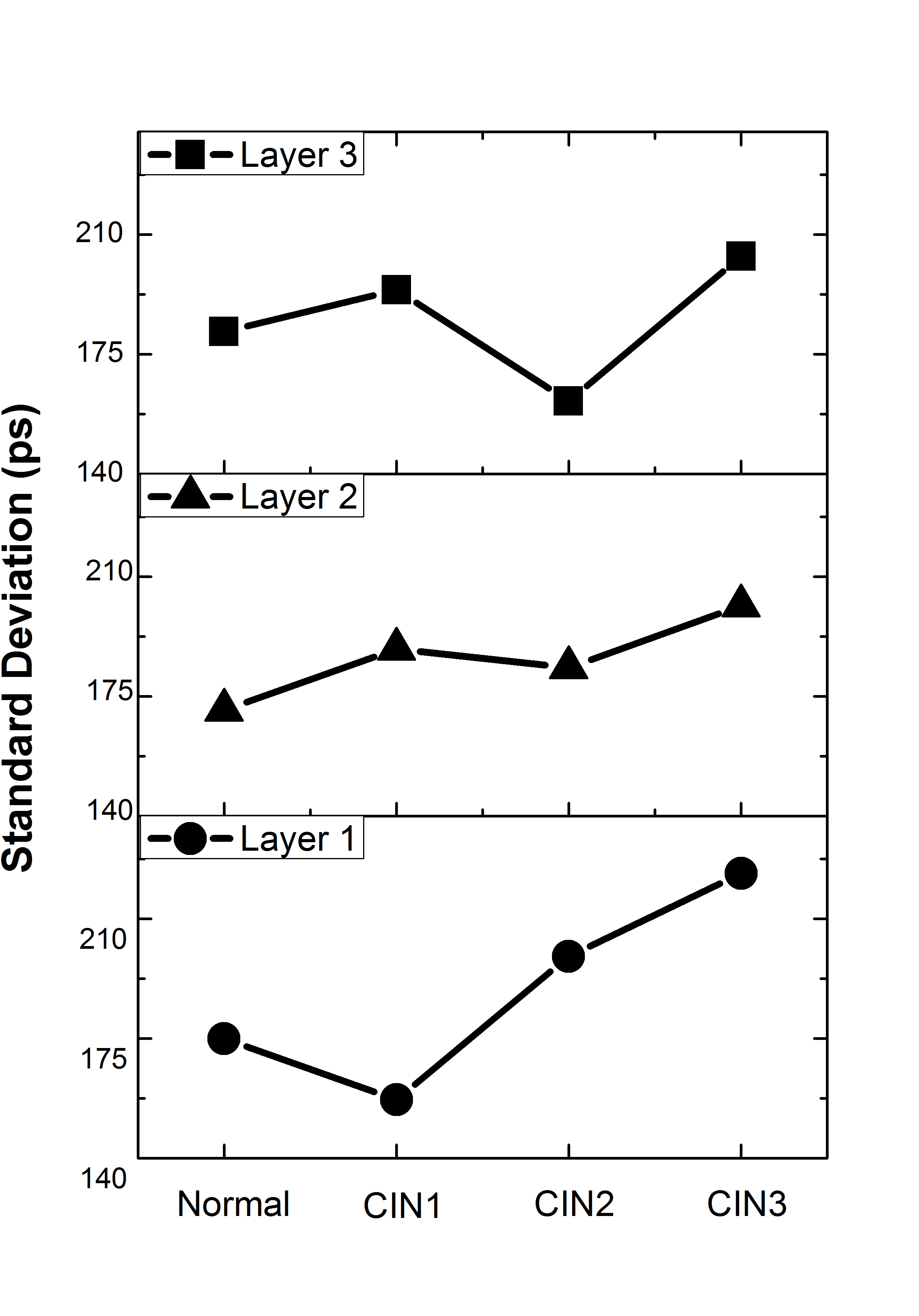

Supplement: S1 Fig — (TIF) [file pone.0125706.s001.tif]

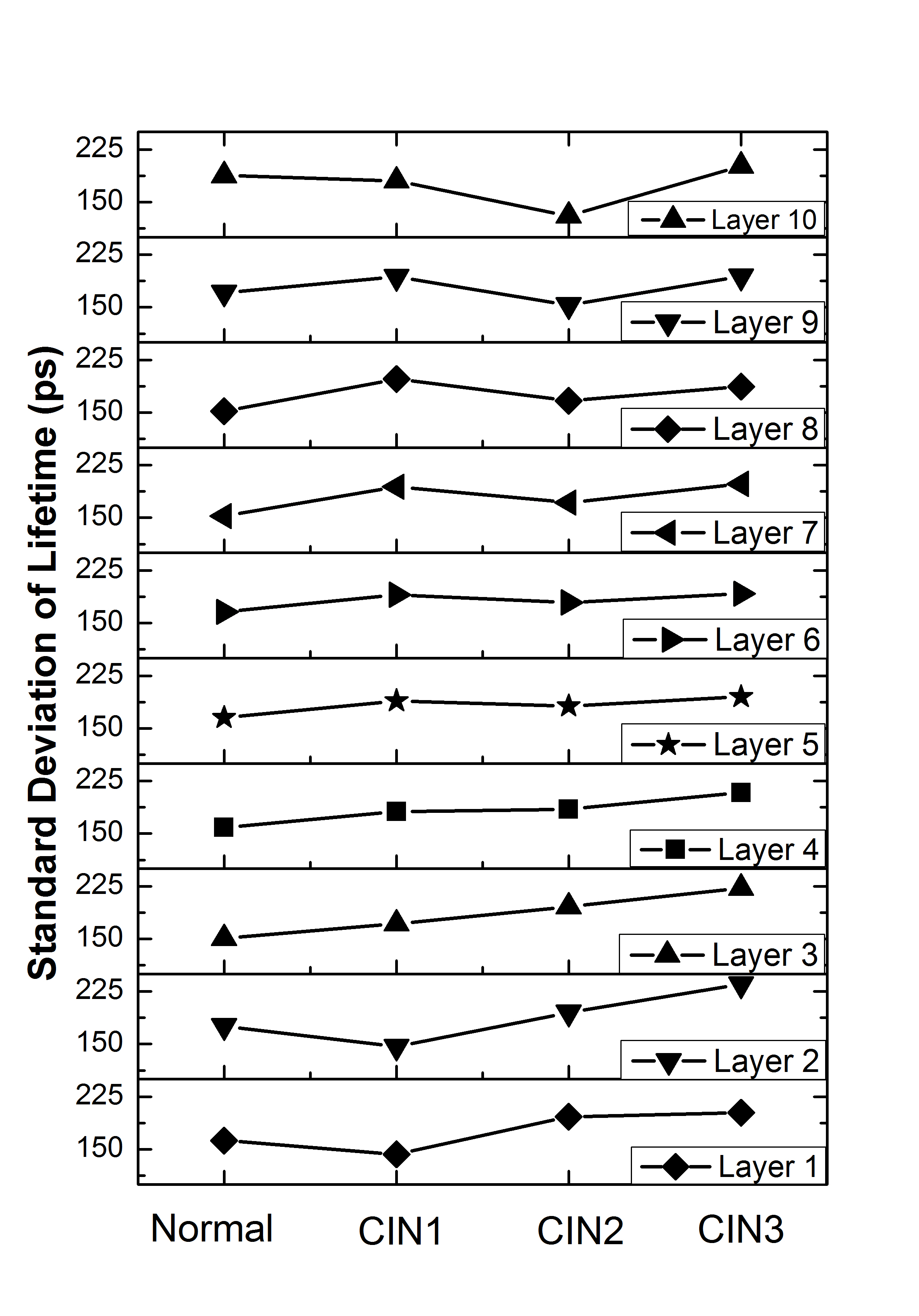

Supplement: S2 Fig — (TIF) [file pone.0125706.s002.tif]

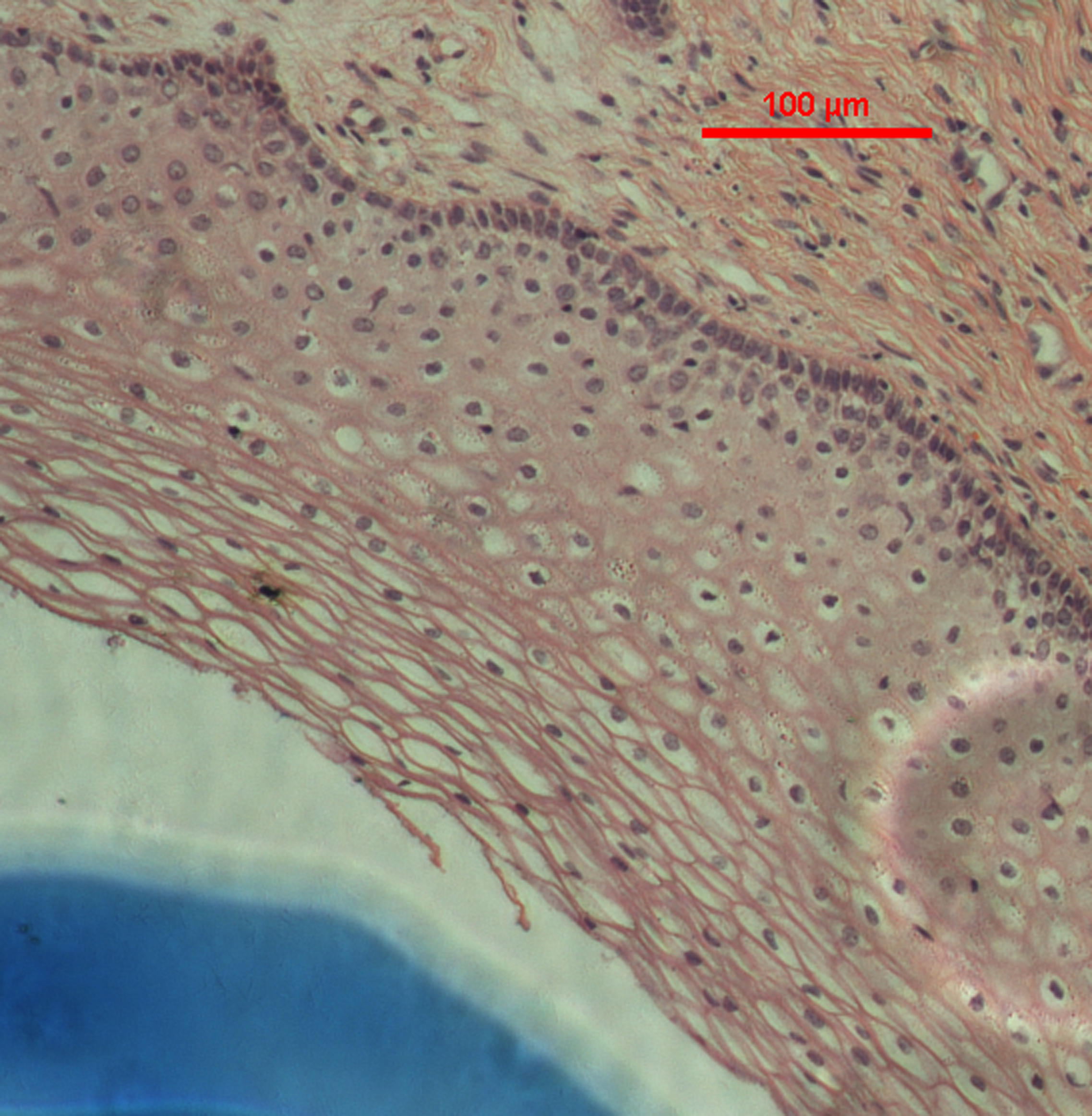

Supplement: S6 Fig — (TIF) [file pone.0125706.s006.tif]
